# Special Issue: State of the Art in Research on Acupuncture Treatment

**DOI:** 10.3390/jcm10245943

**Published:** 2021-12-17

**Authors:** Younbyoung Chae, Myeong Soo Lee, Yi-Hung Chen

**Affiliations:** 1 Acupuncture and Meridian Science Research Center, Kyung Hee University, Seoul 02447, Korea; 2 Clinical Medicine Division, Korea Institute of Oriental Medicine, Daejeon 34054, Korea; drmslee@gmail.com; 3 Graduate Institute of Acupuncture Science, China Medical University, Taichung 40402, Taiwan; yihungchen@mail.cmu.edu.tw

Acupuncture is a medical treatment that involves inserting a needle into the body. Since acupuncture-related techniques and knowledge spread in the West in the 1970s, this treatment has become used and validated for many disorders [1]. Acupuncture stimulation influences target organs via the central and peripheral nervous systems [2]. Functional neuroimaging studies have provided new clues to the neural mechanisms of acupuncture treatment in various diseases [3,4]. Artificial intelligence technology has recently shed light on new approaches to advance acupuncture research [5]. Machine learning can discover connections between diseases and recommended acupoints from large-scale clinical data in the age of artificial intelligence [6,7].

This issue includes 14 articles on the current status of acupuncture research in South Korea, Taiwan, the United States, Poland, and the Czech Republic. It consists of 11 original research articles based on various approaches (e.g., clinical trials, neuroimaging, and machine learning), and 3 review articles that discuss the state of the field and inspire efforts to acquire new insights into acupuncture therapeutics.

The three reviews in this issue outline the present status of acupuncture, cupping therapy, and moxibustion. Using an animal model of post-traumatic stress disorder, a comprehensive review outlines the processes underlying acupuncture [8]. Evidence-mapping methods are used to show the available data in a systematic review of cupping therapy for several illnesses [9]. A bibliometric study reveals the time-based evolution of moxibustion research, as well as the global network of relevant research hubs [10].

The clinical applications of acupuncture and related therapies in this issue include pain management during head and neck cancer radiotherapy [11], chemotherapy-induced peripheral neuropathy in breast cancer patients [12], acute whiplash injury [13], spinal stenosis [14], and poor ovarian function [15]. Resting-state functional neuroimaging studies show the neural underpinnings of acupuncture treatment of primary dysmenorrhea [16] and lower back pain [17]. Furthermore, neuroimaging-based scalp acupuncture suggests dementia therapy locations [18].

Two data-driven approaches reveal acupoint selection and acupoint indication characteristics. Machine learning techniques uncover the commonality and diversity in acupoint selection [19]. Reverse inference and Bayesian factor analyses help discover the specificities of acupoint indications from clinical trial datasets [20]. When artificial intelligence is combined with a wealth of clinical data on acupuncture treatment, connections between diseases and acupoints can be discovered.

We hope that our readers will enjoy this issue of the *Journal of Clinical Medicine*, which extends our knowledge of acupuncture clinical trials and supports the mechanisms of this form of treatment. We believe that with the help of data science technologies, the principles of acupoint selection can be better identified.
